# Teaching Early Reading Skills to Adults With Intellectual Disabilities Using a Support Worker/Family Carer Mediated Online Reading Programme: A Feasibility Randomised Controlled Trial

**DOI:** 10.1111/jar.13332

**Published:** 2024-12-11

**Authors:** Louise D. Denne, Gwenllian Moody, Elinor Coulman, David Gillespie, Kate Ingarfield, Nicholas Manktelow, Corinna F. Grindle, J. Carl Hughes, Zac Taylor, Richard P. Hastings

**Affiliations:** ^1^ Centre for Research in Intellectual and Developmental Disabilities (CIDD) University of Warwick Coventry UK; ^2^ School of Social Policy and Society University of Birmingham Birmingham UK; ^3^ Centre for Trials Research Cardiff University, Neuadd Meirionnydd, Heath Park Cardiff UK; ^4^ School of Educational Sciences Bangor University Bangor Gwynedd UK; ^5^ Achieve Together Bath UK

**Keywords:** adult literacy, feasibility, randomised controlled trial, reading skills

## Abstract

**Background:**

There is a paucity of research into interventions that help people with intellectual disabilities learn to read. This feasibility study examines whether an online reading programme, Headsprout, with additional support strategies and supervision (the intervention), can be delivered by support workers/family carers and the feasibility of conducting a later large‐scale effectiveness trial.

**Methods:**

The study used a 2‐arm randomised controlled trial (RCT) design with an embedded process evaluation using a mixed methods approach.

**Results:**

Thirty‐six adults with intellectual disabilities were recruited. Informed consent and data were obtained remotely. Progression criteria for recruitment, retention, randomisation and usual practice were met; intervention adherence and fidelity were poor. Pressure on support services was a key barrier.

**Conclusions:**

Whilst progression to a large‐scale effectiveness trial was not recommended, the success of conducting an RCT and remotely obtaining informed consent and data from adults with intellectual disabilities opens opportunities for increased participation in research for a currently under‐represented group.

**Registration:** ISRCTN11409097

## 1 Background

Literacy skills are acknowledged to be critical to a person's economic wellbeing and employment prospects, mental and physical wellbeing, family life and taking part in broader societal activities (Teravainen‐Goff et al. 2022; Morrisroe 2014). Low literacy skills are also linked to shorter life expectancy (Gilbert et al. 2018) and, for people with intellectual disabilities, increased co‐morbidity with other healthcare issues compared to the general population (Cooper et al. 2015). Health outcomes are in part related to healthy lifestyle choices. This in turn depends upon health literacy—having access to and being able to understand relevant health information and make informed choices (Nsangi et al. 2017).

‘Literacy’ is a multifaceted and dynamic concept and whilst there are no internationally agreed definitions of the term ‘Literacy’, (UNESCO 2019; Keefe and Copeland 2011) all include the ability to read and write. Reading is also complex involving the interplay of component skills including word recognition and language comprehension with increasing levels of fluency (Scarborough 2001). The National Reading Panel (National Institute of Child Health and Human Development 2000) identifies five critical areas of learning to read: phonemic awareness (the ability to hear and manipulate the sounds in spoken words), phonics (matching the sounds of spoken words with individual letters or groups of letters), fluency (the ability to read with speed, accuracy and proper expression) and vocabulary and text comprehension.

Many individuals with intellectual disabilities have difficulty with learning these basic reading skills and, consequently, have poor literacy skills (van den Bos et al. 2007), potentially limiting their access to critical information relevant to their lives. This secondary impact of intellectual disabilities (Koritsas and Iacono 2011) has implications for the person and those who support them. Key skills that support independence (such as learning to read) have the potential to change the nature of the relationship between adults with intellectual disabilities and their carers—away from a ‘hotel’ model of care in which carers do everything for the person that they are supporting with very little engagement on the part of that person, to ‘active support’ (Toogood et al. 2016) in which persons take an active role in their own lives, prompted and helped when necessary by those who support them.

An option to address a lack of reading skills that has been explored is making information more accessible by using, for example, Easy Read formats (Walmsley 2013). Whilst there are no standards for Easy Read formats, guidelines include the use of simple text, and avoiding passive language and complex tenses. Easy Read formats are effective for some; however, research suggests that this is not always the case (Sutherland and Isherwood 2016). Easy Read is not necessarily tailored to meet individual needs, does not automatically translate into improved understanding (Buell et al. 2020) and, critically, does not teach a person to read (Chinn 2014). Directly developing the reading skills of a person with an intellectual disability may improve independence, quality of life and overall participation in society (van den Bos et al. 2007).

To date, there is very little research into teaching early reading skills to adults with intellectual disabilities and no high‐quality research evidence supported by large‐scale randomised control trials (RCTs) of the effectiveness of strategies to teach adults with intellectual disabilities to read (Alnahdi 2015). More broadly, people with intellectual disabilities are ‘routinely denied opportunities for literacy instruction’ (Keefe and Copeland 2011, pg. 92). This may be partly because of a perception, unsupported by evidence, that it is not possible to teach people with intellectual disabilities whatever their age to read (Kliewer, Biklen, and Kasa‐Hendrickson 2006), and that the ability to learn to read plateaus in adults with intellectual disabilities (Moni, Jobling, and Kraayenoord 2007; Morgan, Moni, and Jobling, 2004). Recently, however, studies have shown that it is possible to teach reading to people with intellectual disabilities (Grindle et al. 2021, 2013; O'Sullivan, Grindle, and Hughes 2017; Tyler et al. 2015; Allor et al. 2010) with appropriate strategies such as phonics teaching and opportunities to practice learning to decode text. In addition, although learning may progress more slowly, it is possible for adults with intellectual disabilities to continue to learn to read into adulthood (Browder and Xin 1998; Pershey and Gilbert 2002).

The Grindle and Tyler studies referenced above used Headsprout Early Reading (HER) an established online reading programme initially developed in the United States of America for typically developing children aged 4 to 8 who were struggling to read in mainstream education. HER is based on 4 years of research and development (Layng, Twyman, and Stikeleather 2003) and uses an understanding of effective instructional processes to ensure reading success for all learners. The programme incorporates sight reading and systematic instruction within the early skills involved in decoding identified by the National Reading Panel noted above. HER works at the pace of the learner, giving the opportunity to practice decoding and sounding out and understanding words through 80^1^ online episodes. An RCT in the USA showed HER to be effective with typically developing children (Huffstetter et al. 2010).

Pilot research run in the UK with children attending special schools and resource units aged between 5 and 19 has suggested that HER along with supplementary support strategies specifically targeting additional needs such as activities where children are having difficulty with attending, motivation or specific concepts (e.g., negation), can be effective for children with intellectual disabilities (Grindle et al. 2021, 2013; Roberts‐Tyler, Hughes, and Hastings 2020; Tyler et al. 2015).

In an innovative study in England, O'Sullivan, Grindle, and Hughes (2017) used HER in a secure hospital setting to teach basic reading skills to adults with a mild intellectual disability. As with the studies delivered in schools, the intervention included supplementary activities targeting each participant's additional needs; although no adaptations to the HER programme were needed despite it having been developed for neurotypically developing children. The study showed improved decoding skills critical to reading and demonstrated the feasibility of using the HER intervention with an adult population (O'Sullivan, Grindle, and Hughes 2017). However, the intervention was delivered by trained researchers in a secure setting in which the intervention could be delivered with fidelity. It is unknown whether the feasibility of running the programme under these conditions translates to typical community and care settings for adults with intellectual disabilities.

The current feasibility study was funded by the National Institute of Health Research under its first programme for social care research. Its aim was to examine whether the intervention (HER, with additional support strategies and supervision), can be delivered successfully by community support workers/family carers, whether learning to read has any impact on health and social care measures and whether it would be feasible to conduct a later large‐scale effectiveness trial to explore its effectiveness. Consistent with feasibility studies of this nature (Orsmond and Cohn 2015) there were a number of feasibility questions: (1) what are the most effective recruitment pathways to identify adults with intellectual disabilities who are interested in learning to read, and who have support workers/family carers willing to support them; (2) can sufficient provider organisations/families be recruited to participate; (3) are adults with intellectual disabilities and their support workers/family carers willing to be randomised within the context of an RCT; (4) can support workers/family carers deliver the intervention with a high degree of fidelity; (5) what proportion of adults with an intellectual disability and support workers/family carers complete the intervention (all 80 episodes of HER) before the 6‐month follow‐up; (6) what proportion of adults with an intellectual disability and their support workers/family carers are retained in the research study to the 6‐month post‐randomisation follow‐up; (7) what does usual practice consist of for adults with intellectual disabilities in support of their reading in social and family care settings; (8) what health/social care/quality of life and reading outcomes measures best address the aims of the study; and (9) what is the feasibility of collecting health‐related quality of life data and service use data for adults with intellectual disabilities?

## 2 Methods

Full methods are detailed by (Moody et al. 2022) in the published protocol. A brief description follows.

### 2.1 Study Design

The study was a 2‐arm feasibility RCT with an embedded process evaluation using a mixed methods approach based on the Medical Research Council (MRC) framework (Craig et al. 2008). Randomisation to the intervention (HER) or control groups (usual practice) was 1:1 using randomly permuted blocks (block sizes of 2 and 4), stratified by setting type (family home vs. other social care setting). To ensure allocation concealment and preserve blinding of the Trial Statistician, randomisation was maintained centrally in the Centre for Trials Research at Cardiff University (removed for blinding purposes) by a Statistician not involved in statistical analysis and block size and stratification details were not disclosed to those involved in recruitment of participants.

Semi‐structured qualitative interviews with both participants and support workers or family carers, and discussions with an Advisory Group formed from the Public and Patient Involvement (PPI) team, were used to inform the process evaluation. Interviews were also conducted with the support workers and family carers of potential participants who had expressed an interest in, but subsequently declined, to take part in the study.

### 2.2 Sample Size

The study aimed to recruit 48 individuals (randomising 24 per trial arm). As this was a feasibility study, the purpose was to provide estimates of key parameters for a future trial rather than to power the current study to detect statistically significant differences.

### 2.3 Study Population

Participants were eligible for trial if they were adults administratively defined as having an intellectual disability; had capacity to give informed consent (as reported by their support worker/family carer but also verified prior to baseline data collection at recruitment using a protocol developed for the study); had sufficient competence in understanding English to access HER (assessed using a HER placement assessment); an ability to vocalise; access to internet‐enabled technology; basic mouse skills, or the capacity to be taught; and skills support from a support worker/family carer who could read and were willing to support them. Adults with intellectual disabilities with severe visual impairments with no opportunity for correction were excluded because HER does not provide adaptations for the visually impaired.

### 2.4 Reading Intervention

The intervention included an established online reading programme HER; an accompanying support manual detailing supplementary support strategies tailored for people with intellectual disabilities such as additional activities to help with specific areas of difficulty; training for support workers and family carers involved in the delivery of the intervention, and fortnightly supervision from a member of the research team for its duration.

The intervention was developed with the help of PPI representatives including three adults with intellectual disabilities, three support workers and one social care provider service manager. PPI input included consultation on the support manual for support workers/family carers and the support worker/family carer training; developing an intervention logic model; the co‐production of two new measures: a reading self‐concept assessment for adults with an intellectual disability and a supporting reading self‐efficacy assessment for support workers/family carers; advising on the presentation of existing outcome measures; and piloting the online recruitment process.

Following randomisation and before accessing HER, support workers and family carers for intervention group participants were offered a half‐day tailored training session delivered by CG (removed for blinding purposes), one of the clinical leads for the study, and given the support manual. Training was offered face‐to‐face prior to the COVID‐19 pandemic and online once the study resumed. The intervention was expected to last for approximately 6 months post‐randomisation. The HER programme consisted at the time of 80 online episodes delivered in sessions of approximately 20–25 min. Participants were asked to try to complete at least three HER sessions of 20–25 min per week, recommended by HER but also shown in previous studies to be essential for skills development (Grindle et al. 2021). Throughout the intervention, support workers and family carers were offered bi‐weekly telephone support and supervision including trouble shooting, sign pointing to additional resources in the support manual and further suggestions to help with specific areas of difficulty. The researcher offering this supervision was experienced in using HER with learners with an intellectual disability, had access to HER and was able to monitor individuals' progress and provide tailored support.

### 2.5 Procedure

Ethical approval for the study was granted in December 2019 by the NHS Health Research Authority, London—Camberwell St. Giles Research Ethics Committee (reference number 19/LO/1784).

Participants were invited to participate using multiple pathways (emails to service providers, support and advocacy groups, social media distribution and on‐line meetings with social groups) in a single study site (the University).

Face‐to‐face recruitment in participants' home settings began in January 2020 was paused in March 2020 because of the COVID‐19 pandemic and resumed in January 2021 with adaptations to the recruitment process to facilitate online consent and data collection. Recruitment ended in May 2021. Participants were recruited from family homes, independent living and small group settings. Informed consent was obtained from both participants and their support workers or family carers, and baseline data were collected prior to randomisation.

Post‐intervention data were collected 6 months post‐randomisation. All participants and their support workers or family carers in the intervention group were invited to take part in semi‐structured interviews once they had completed the post‐intervention data collection. The researcher collecting both baseline and post‐intervention data was blind to participants' group allocations during data collection. Once post‐intervention data collection was complete, however, participants were asked which group they were in so that those in the intervention group could be invited to take part in semi‐structured qualitative interviews. All participants received usual practice in relation to their reading support. Control group participants were given the support manual, access to a generic pre‐recorded training module and access to HER at the end of the study.

To facilitate retention, adults with an intellectual disability and support workers/family carers were offered a £20 and £10 voucher respectively as a thank you for baseline and follow‐up data collection. Those participating in interviews were also offered an additional £20 voucher.

### 2.6 Measures

#### 2.6.1 Trial Feasibility Outcomes

The feasibility of conducting a large‐scale effectiveness trial was the primary outcome in this study. The following feasibility outcomes, measured using a combination of descriptive, quantitative data and qualitative data, informed the decision regarding progression to a later large‐scale effectiveness trial: (1) participant recruitment including the percentage of participants who consented to and who were willing to be randomised to the trial, recruitment rate and most effective pathways and recruitment of support organisations and family carers willing to support participants through the intervention; (2) participant retention at 6‐months post‐randomisation; (3) the percentage of participants in the control arm who received an alternative structured reading programme between baseline and 6‐month follow‐up (i.e., contamination); (4) fidelity to the HER component of the intervention; (5) adherence to the intervention; (6) exploration of the health/social care/quality of life and reading outcomes measures that best addressed the aims of the study; and (7) the feasibility of collecting health‐related quality of life data and service use data. Data sources included a recruitment and retention log, discussions with the study Advisory Group as well as other data sources described below.

#### 2.6.2 Participant‐Reported and Assessed Outcome Measures

At baseline, participants provided demographic data (gender, year of birth and support and living arrangements).

The Dynamic Indicators of Basic Early Literacy Skills (DIBELS) (Good and Kaminski 2002) was used to assess decoding skills involved in reading. A validated measure (Hintze, Ryan, and Stoner 2003) consists of 5 one‐minute fluency assessments including letter naming, phonemic identification, decoding nonsense words, reading words and reading script. Normally administered face‐to‐face, the DIBELS was adapted for this study to be delivered remotely via Microsoft Teams because of COVID‐19.

A reading self‐efficacy measure (and carer efficacy in supporting the person to read) was used to assess three domains: attitudes towards reading, perceptions of difficulty with reading, and perceptions of competence in reading. The measure was based on the reading self‐concept scale (RSCS) by Chapman and Tunmer (1995) and adapted with the help of PPI including reducing the number of items from 30 to six and changing statements from a school based to an adult setting context. The adapted version consists of six short statements (two for each domain) read out to each participant. Participants were asked to use a three‐point scale: ‘always’, ‘sometimes’ or ‘never’ that best corresponded with their views on reading. The scale was also presented with three, two and one stars as a visual alternative based on PPI feedback that stars were preferable to other symbols such as smiley faces.

The EQ5D‐3L, a standardised measure to describe and value health‐related quality of life across five domains: mobility, self‐care, usual activities, pain/discomfort and anxiety/depression, was used to assess participants' views of their health state (Kind, Brooks, and Rabin 2005). Each domain is presented with three options: no problems, some problems or extreme problems. Statements were read out loud to participants who were asked to indicate the option that best described their current health state.

The Personal Well‐Being Index Intellectual Disability version (Cummins and Lau 2005) was used to measure satisfaction across seven domains: standard of living, health, life achievement, personal relationships, personal safety, feeling part of the community and future security. The index is completed by the person with an intellectual disability using a 10‐point Likert scale. The manual offers options for participants who may struggle with a 10‐point scale. For this study statements were read out and a four‐point scale was used: ‘not at all happy’, ‘a little bit happy’, ‘happy’, ‘very happy’. For consistency, the visual alternative of one to four stars was also offered.

The Client Service Receipt Inventory (CSRI) from a previous intellectual disability study (Beecham and Knapp 1992; Jahoda et al. 2017) was used to examine the feasibility of collecting data for a future health economics analysis from family carers and support staff. It collects information on service utilisation, income, accommodation and other cost‐related variables in relation to a person's support.

Intervention adherence (including fidelity) data were collected from data provided by HER and from the bi‐weekly supervision sessions. Adherence was defined as percentage of support workers and family carers attending the training prior to starting the HER programme, percentage of participants starting at least one session per week for a total of 20 weeks, based on whether they logged into the session and attendance by the support worker or family carer at the bi‐weekly support sessions. Fidelity as measured by HER was defined as repeating episodes when necessary. HER requires learners to complete each episode with 80% accuracy (mastery) before moving on to the next episode.

### 2.7 Data Analyses

#### 2.7.1 Statistical Analysis

As this was a feasibility trial, no formal hypothesis testing took place. Descriptive statistics were reported as means and standard deviations or medians and interquartile ranges, as appropriate and categorical data were reported as frequencies and proportions. All data were reported both overall and by study arm. Feasibility outcomes were estimated alongside 95% confidence intervals. The findings are reported in line with the CONSORT extension for pilot and feasibility studies (Eldridge et al. 2016).

#### 2.7.2 Economic Analysis

Whilst the study did not include a formal economic analysis, the feasibility of collecting health and economic data (quality of life and CSRI) was assessed with a view to informing a future trial.

#### 2.7.3 Qualitative Analysis

Interview data were analysed using thematic coding as detailed by Gibbs (2007). Interviews were transcribed (audio into text) verbatim without annotation by a study administrator. Coding was done by the first author. Initially, ‘line by line’, ‘open’ coding (i.e., without starting with an anticipated list of codes) was used, whereby all content within each line was assigned a code identified by the researcher. The process used was inductive. This attempts to mitigate against subjectivity on the part of the researcher who may be tempted to seek out codes based on pre‐conceived ideas of what the data might contain although in practice thematic coding always involves the researcher as an active participant in the generation of themes. Similar codes were grouped together, and themes constructed to reflect participants' experiences of the intervention and taking part in the research process. A sample of interviews were independently coded by the second author as a reliability check.

### 2.8 Data Synthesis

The process evaluation integrated the respective data sources and focused on the key feasibility questions. A traffic light system (Eldridge et al. 2016): green indicating ‘go without any modification necessary’; amber indicating ‘potential proceed to large‐scale effectiveness trial, remedying early issues’; red indicating ‘Stop’ was used to inform the recommendation about whether to progress to a large‐scale effectiveness trial (see Table 1). The responsibility for that decision lay with the Study Steering Committee—an independent group chaired by an expert in intellectual disability research and in trials and including an independent intellectual disabilities expert/clinician, independent statistician and a family carer representative (family member of an adult with intellectual disabilities).

**TABLE 1:** Summary of traffic light progression criteria.

Feasibility questions	Traffic light criteria
**Participant recruitment:** % of participants approached, and who are eligible, consent to the study (and thus are willing to be randomised)	Green ≥ 50% Amber 30% ≥ < 50% Red < 30%
**Individual randomisation possible:** (% of total number of settings in which more than one participant is eligible and willing to take part) (NB. Amber/red here may lead to a proposal for a cluster randomised design)	Green ≤ 20% Amber 20% > ≤ 40% Red > 40%
**Rate of recruitment:** % of recruitment target (48 participants) are recruited within the study recruitment period	Green 100% Amber 70% ≥ < 100% Red < 70%
**Participant retention:** % of participants retained 6 month follow‐up data collection timepoint	Green 75% < > 100% Amber 50% ≥ < 70% Red < 50%
**Usual practice:** % of participants in the UP arm of the study who receive an alternative structured programme designed to teach them to read between baseline and 6 month follow‐up	Green ≤ 30% Amber 30% > ≤ 50% Red > 50%
**Fidelity:** Self‐rating forms indicate % of READ‐IT manual components have been met both across and within sessions.	Green 70% < > 100% Amber 50% ≥ < 70% Red < 50%
**Adherence:** % of participants and their support workers/family carers who adhere to the READ‐IT programme (attend training, complete 80 episodes within 20 weeks, meet adherence criteria built into HER programme)	Green > 70% Amber 50% ≥ < 70% Red < 50%

## 3 Results and Discussion

### 3.1 Participants

**FIGURE 1:**
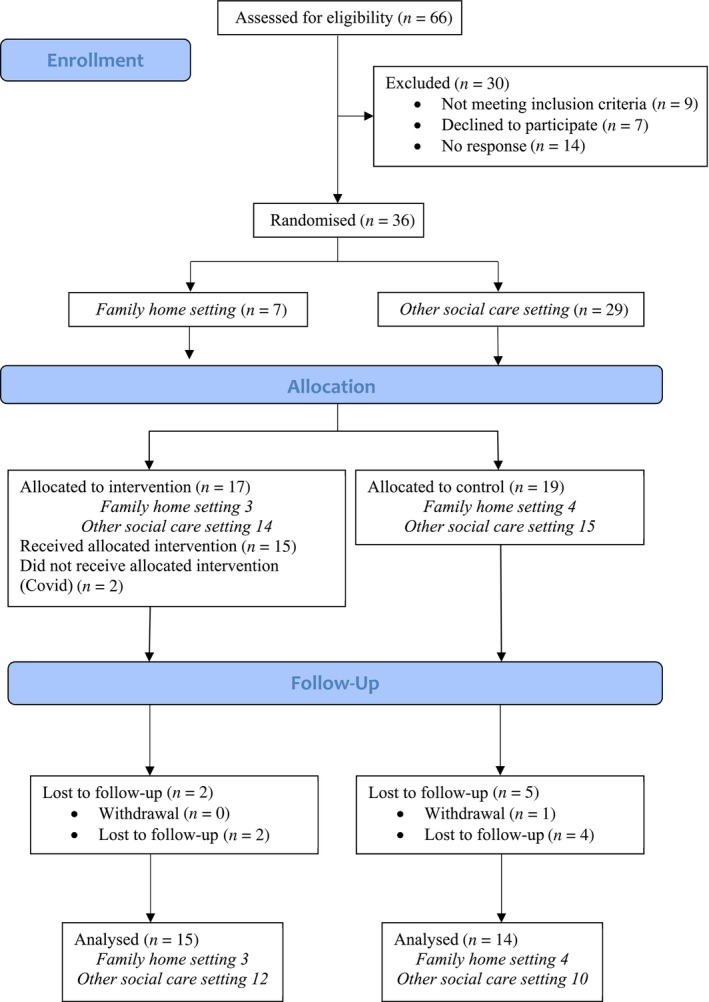
Study CONSORT Diagram.

Thirty‐six adults with an intellectual disability were recruited (Figure 1). Participants ranged in age from 21 to 59 years old with a mean of 47 years (IQR 29.6, 46.9). The majority (*n* = 22, 61%) were male. Seven (19%) participants were supported by family carers. Twenty‐nine (81%) participants were supported in a social care setting and seven (19%) in a family home setting. Five (14%) participants lived in a setting in which more than one person might be interested but eligibility for other individuals was not assessed (Table S1). Two support workers, one family carer and two adults with a learning disability took part in the interviews.

### 3.2 Feasibility of Recruiting Eligible Participants, Recruitment Rates, Most Effective Recruitment Pathways and Acceptability of Research Design

Of 66 participants who expressed an interest in participating, 57 were screened and 36 consented (54% of those expressing interest), completed baseline measures, and were randomised. This represents 75% of the target recruitment number (48).

Recruitment was primarily via written e‐mail communication sent to 181 contacts including provider organisations, family carer organisations, support groups and local authorities. Eighty‐six percent of participants recruited were contacted through their provider agency; 11% through ‘other’ (predominantly family support groups); and 3% via social media (mail and text messages). Twenty‐three provider organisations out of a total of 181 contacted expressed an interest in the study, and 20 were involved in supporting those adults recruited.

The recruitment process was time‐consuming. Administrative bureaucracy within some services required checking up the management hierarchy; at least one organisation had an internal research governance process that had to be completed before recruitment could progress. Primarily however the need to go through gate keepers (dependent therefore on their availability, responses to texts/emails) built in additional time between contacting service providers and direct contact with potential participants (pre‐pause average 32 days, post‐pause average 49 days) and time then taken to set up initial recruitment meetings. In addition, the move to remote recruitment because of COVID‐19 involved separate sessions for obtaining informed consent and baseline data collection. The average number of days from initial direct contact to baseline data collection was 49 days ranging from 11 to 130 days. A discussion with the study Advisory Group post‐recruitment suggested that there may be more effective ways of recruiting (e.g., accessing and talking to adults with intellectual disabilities directly using verbal communication as opposed to using written material that depended on ‘gate keepers’). Examples given included distributing podcasts or videos and attending local social events.

One support worker and two family carers who had expressed an interest in the study but subsequently declined to take part were interviewed to help understand potential barriers to recruitment. All three reported that they thought that learning to read is an important skill and were interested in finding a way of achieving this despite not signing up. Reasons given for not going on to participate in the study included logistical issues (including not enough time, no suitable venue, no internet access and a lack of support from the provider organisation), personal issues, and the fact that this was a research study rather than the offer to participate in a stand‐alone reading programme.

Facilitating factors for taking part in the research were assessed in qualitative interviews with three support workers/family carers and two adults with an intellectual disability. The primary motivation for taking part was a desire to learn to read. Linked to this was the value attached to reading as a useful skill for adults with intellectual disabilities and a current lack of resources available to help adults with intellectual disabilities learn to read.

Discussions with the Advisory Group suggest that family carers/adults with intellectual disabilities may not be willing to be supported to learn to read by family members: ‘I do not want my mum to teach me—I want to be independent’ (Advisory Group member). The Advisory Group noted however that some family carers may be willing to support others within the community on a voluntary basis.

Nineteen participants were randomised to the control group and 17 to the intervention group. During the recruitment process, no participant or support worker/family carer cited a concern with randomisation as a reason not to participate, suggesting that an individually randomised design was feasible.

### 3.3 Retention Through 6 months' Post‐Randomisation Follow‐Up Data Collection

Thirty‐one of 36 participants were retained to follow‐up (Figure 1). This represents an 86% (95% CI:71%, 94%) retention rate. Of the participants lost, there was one death (unrelated to the intervention), two participants lost to follow‐up subsequent to the study interruption as a result of the COVID‐19 pandemic, one was unavailable for post‐testing due to ill‐health, and one did not respond. All the participants who were lost to follow up were supported by social care providers.

### 3.4 Adherence, Reach and Fidelity of Implementation of the Intervention

**FIGURE 2:**
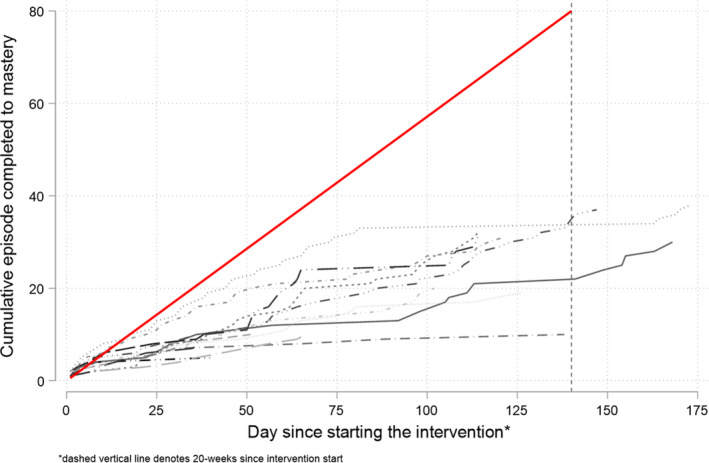
Cumulative episode completed to mastery per participant.

Of the 17 participants allocated to the intervention arm, 15 started the intervention (88%, 95% CI: 66%–97%). No participant completed all 80 episodes in the version of HER shared with participants. No participants appeared to start at least one online episode per week for a total of 20 weeks (Figure 2). However, online episodes may have been supplemented with offline sessions/activities suggested in the support manual. These were not recorded as part of the study.

The timetabling of sessions was raised during the bi‐weekly supervision sessions. The most commonly cited difficulties were staff absence/shortages and difficulties of scheduling delivery time because of changing staff and changing rotas: 4 out of 15 (27%) original support workers left the service part way through, and eight out of 11 (73%) original support workers cited staff shortages and being too stretched to deliver the intervention consistently. Two out of the 17 support workers/family carers supporting participants in the intervention group (12%) cited behavioural issues as an additional barrier to implementation. These were not prompted by attempts to engage with the intervention, rather participants reported defined periods of unsettled behaviour due to other factors such as changes to routines, staff changes and the COVID‐19 pandemic.

Of those who started the intervention and for whom data on episode completion were available, the median number of episodes completed to mastery was 20 (IQR: 10–31), with the total number of episodes completed to mastery (for those who started the intervention) ranging from 2 to 38. Similarly, over the first 20 weeks (anchored to the date of the first session), the median number of episodes completed to mastery for the 14 participants with episode data was 20 (IQR: 10–31, min = 2, max = 35).

At least one HER episode was repeated by 12/14 participants (86%, 95% CI: 60%–96%), with the three most repeated episodes being 23 (repeated by six participants), 15 (seven participants) and 21 (five participants). One participant progressed onto a new episode without having achieved mastery. The remaining 13 participants either always achieved mastery (*n* = 5), did not progress onto a new episode without first achieving mastery (*n* = 5) or progressed onto a new episode but subsequently (i.e., not immediately repeating) repeated the episode during which mastery was not achieved (*n* = 3).

A condition of starting the programme was that all support workers/family carers be trained at the start of the intervention. Carers associated with 16 participants received training (94%, 95% CI: 73%–99%), with two receiving refresher training (necessary due to a pause related to the COVID‐19 pandemic). The delivery of the intervention was dependent on the trained support worker/family carer. Therefore, intervention delivery was negatively impacted if support workers changed jobs or shift patterns changed. Care organisations were given the opportunity to train new or back‐up support workers using recorded online training sessions, but this was not taken up.

Carers associated with 12 of the 17 intervention group participants engaged in the bi‐weekly supervision and mentoring process (71% [95% CI: 47%–87%]). This was 80% (95% CI: 55%–93%) of the 15 participants starting the intervention. Of those carers engaging in the bi‐weekly telephone calls, the median percentage of calls answered on the arranged date and time was 82% (IQR: 65%–93%, range: 28%–100%).

It was clear that staffing problems were partly a result of the COVID‐19 pandemic, but discussions with the Advisory Group suggest that even pre‐pandemic care providers were under pressure and continuity of service provision was difficult (LGA 2022). Interviews with two support workers and one family carer also cited difficulties with scheduling the intervention into daily activities:

And we could have really focused on it. But obviously that… that's not… you know, how life normally is. So, do when she came home at weekends… and we managed two or sometimes three. (Family carer).

No, no difficulties. The only thing was, if myself or [ph] wasn't here… then it was a bit more difficult for him, because the other staff really didn't know what to do. (Support worker).

Participants cited technical difficulties during implementation (five participants had initial difficulties logging in/accessing online resources, one participant had difficulties moving on after a particular episode and one participant cited difficulty using Microsoft Teams for training), but these were isolated and resolved in the mentoring sessions. Just one support worker raised concerns about the appropriateness of the intervention for the person that they were supporting:

it's nothing to do about the programme. I think the programme is fine, is very clear … the issue was … the way of the programme trying to teach is not compatible with his way, his, his brain works. It just doesn't.

### 3.5 Usual Practice for Adults With Intellectual Disability in Support of Their Reading in Social and Family Care Settings

One participant of 14 control participants who completed follow‐up (7%; 95% CI: 1%, 32%) reported receiving ‘Some small bits of reading as part of adult education courses—not main focus of these—very small’. Otherwise, there was no support of reading provided as usual practice.

### 3.6 Feasibility and Acceptability of Proposed Outcome Measures for a Definitive Trial, Including Resource Use and Health‐Related Quality of Life Data

There were no reported difficulties in administering the proposed outcome measures (Tables S2–S18) and no missing data for the DIBELs, or reading self‐concept measures, suggesting that it would be feasible to include these in a definitive trial.

Although no formal economic analysis was planned, the feasibility of collecting health‐related quality of life and service use data was assessed using the EQ5D, the CSRI and personal wellbeing index. There was no missing data for the EQ5D. Five participants did not answer one question on the personal wellbeing index at follow‐up: ‘How happy do you feel about how things will be later in your life?’ although this question was answered at baseline. There was occasional refusal to answer optional questions about use of psychotropic medication in the CSRI (‘cannot see relevance’).

### 3.7 The Feasibility of Implementing HER Within a Definitive RCT: Assessment of Criteria to Progress to a Large‐Scale Effectiveness Trial

Progression criteria were used by the Study Steering Committee (SSC) to inform the decision to progress to a large‐scale effectiveness trial. Although the primary progression criteria around recruitment and intervention mechanisms were green or amber, progression criteria around intervention implementation were not met (Table 2). The SSC concluded that it was not possible to continue to a large‐scale effectiveness trial at this stage.

**TABLE 2:** Results of progression criteria.

Measurement	n/N	Proportion	95% CI
Participant recruitment	36/57	63.2%	50.2%, 74.5%
Individual randomisation possible	5/36	13.8%	6.1%, 28.7%
Rate of recruitment	36/48	75.0%	61.2%, 85.1%
Participant retention	31/36	86.1%	71.3%, 93.9%
Usual practice	1/14	7.1%	1.3%, 31.5%
Adherence and fidelity	0/17	0.0%	n/a

## 4 Conclusions

The study's primary aim was to assess whether HER with additional support can be delivered successfully by community support workers/family carers. It is clear from the results that whilst it is possible to deliver certain elements of the intervention (attendance at training, attendance at the bi‐weekly supervision sessions) full adherence, and delivering HER with fidelity, was not achieved. This was not because of a lack of commitment on the part of participants or family carers and support staff; nor was it because of a lack of motivation on the part of participants to use the programme or to learn to read, or their support workers and family carers to support them through the intervention. Just one support worker questioned the appropriateness of the intervention for the person they supported. Rather it reflected the widely acknowledged (LGA 2022) pressures on the care sector in the United Kingdom at the time. Despite best intentions, it was not possible to provide consistency and continuity of support in respect of the intervention and, with competing priorities, difficult to schedule delivery time into daily activities. Stressors on the care sector were exacerbated by the COVID‐19 pandemic. This meant that care organisations could not ensure that the support person who had agreed to participate in the study would be available for the full length of the intervention and would be timetabled to support the participant as recommended by previous studies (Grindle et al. 2021) more than once a week or on successive weeks. Support workers adhered to the intervention by attending bi‐weekly sessions, often in their own time, but were unable to do HER sessions at least once a week with the adult that they were supporting.

Discussions with the Advisory Group suggest that any future delivery is likely to be a hybrid of options with some core characteristics. Any reading intervention should be addition to, and not part of, day‐to‐day support (i.e., extra resources need to be made available as day‐to‐day support is currently stretched); and combined with preferred social activities and tailored to the individual (e.g., make an ‘outing’ of it, going to a café, social group, etc.). The recruitment of a core team of trained readily available staff (and potentially volunteers) in identified service provider centres/libraries who focus exclusively on reading interventions could be considered. These approaches could be evaluated initially using single‐case experimental designs to allow some exploration of the impact of the heterogeneity of the participant population.

More encouraging findings from the current study included: successful recruitment and retention in a randomised trial in social care settings, and the demonstration that it is possible to remotely obtain both informed consent from adults with intellectual disabilities, and data including health‐related economic data. This opens opportunities for increased participation in research for a currently under‐represented group. Thus, carrying out randomised trials of educational interventions in community care settings may be feasible with interventions that are feasible to deliver.

## Author Contributions

L.D.D. managed the trial, conducted qualitative analysis, wrote up results for publication and prepared the article for publication. G.M. managed the trial and contributed to article writing. E.C. supervised the Cardiff University team and provided expertise in trial design and management and contributed to article writing. D.G. managed the statistical analysis and wrote up the results for publication. K.I. carried out the statistical analysis and contributed to article writing. N.M. carried out quantitative and qualitative data collection and contributed to article writing. C.F.G. provided expertise in the HER intervention and conducted the training. J.C.H. provided expertise in the HER intervention. Z.T. led the PPI component of the trial. R.P.H. provided expertise in trial design and management, mentorship for L.D.D. and contributed to article writing. All authors contributed to article writing. All authors read and approved the final manuscript.

## Ethics Statement

Ethical approval for the study was granted in December 2019 by the NHS Health Research Authority, London—Camberwell St Giles Research Ethics Committee (reference number 19/LO/1784). The study has been performed in accordance with the ethical standards laid down in the 1964 Declaration of Helsinki and its later amendments. All participants gave their fully informed consent prior to their inclusion in this trial. All details that might disclose the identity of participants have been omitted.

## Consent

All participants gave their consent for their pseudonymised data to be published in a research article.

## Conflicts of Interest

The authors declare no conflicts of interest.

## Supporting information


**Data S1.** Supporting Tables.

**Data S2.** CONSORT 2010 checklist of information to include when reporting a randomised trial.

## Data Availability

The data that support the findings of this study are available from the corresponding author upon reasonable request.

## Endnotes

^1^
HER now consists of 100 episodes.
