# Supplementary material for: Teaching Early Reading Skills to Adults With Intellectual Disabilities Using a Support Worker/Family Carer Mediated Online Reading Programme: A Feasibility Randomised Controlled Trial
Source: J Appl Res Intellect Disabil. 2024 Dec 11;38(1):e13332. doi: 10.1111/jar.13332 (PMC11635184; doi:10.1111/jar.13332)
Supplement: Supplementary file 1 — Data S1. Supporting Tables. [file JAR-38-e13332-s001.docx]

**READ-IT Supplementary Tables**

Table S1: *Participant characteristics*

| **Characteristic** | **Number** |
| --- | --- |
| **Age** |  |
| Mean | 47 |
| Range | 21-59 |
| Median | 34 |
|  |  |
| **Gender** |  |
| Female | 14 |
| Male | 22 |
|  |  |
| **Setting** |  |
| Family home | 7 |
| Community setting or supported living | 29 |
|  |  |
| **Support type** |  |
| Family member | 5 |
| Support staff | 31 |

Table S2: Baseline CSRI 1-3

|  | **Total** | **Intervention (n = 17)** | **Control (n = 19)** |
| --- | --- | --- | --- |
| **Total – N (%)** | 36 (100.0%) | 17 (47.2%) | 19 (52.8%) |
| **Age – Median (IQR)** | 34.1 (29.6, 46.9) | 34.6 (32.7, 47.4) | 31.4 (27.6, 46.4) |
| **What is the job title of the support worker?** |  |  |  |
| Support worker/carer | 20 (55.6%) | 9(52.9%) | 11 (57.9%) |
| Registered/Service/Home/Locality Manager | 6 (16.7%) | 2 (11.8%) | 4 (21.1%) |
| Team/eam leader | 4 (11.1%) | 3 (17.6%) | 1 (5.3%) |
| Senior social care worker | 1 (3%) | 1 (6%) | 0 (0%) |
| Retired | 2 (6%) | 1 (6%) | 1 (5%) |
| Family Carer | 3 (8.3%) | 1 (5.9%) | 2 (10.5%) |
| **What is the service user’s usual place of residence?** |  |  |  |
| Staffed group home or supported living | 28 (78%) | 14 (82%) | 14 (74%) |
| Family home | 6 (17%) | 2 (12%) | 4 (21%) |
| Adult family placement | 2 (6%) | 1 (6%) | 1 (5%) |
| **In the last 3 months, has the service user done any paid work?** |  |  |  |
| No | 31 (86%) | 14 (82%) | 17 (89%) |
| Yes | 5 (14%) | 3 (18%) | 2 (11%) |
| **If yes, what was/is the service users occupation?** |  |  |  |
| Office / Clerical worker (e.g., office assistant, photocopying) | 1 (20%) | 1 (33%) | 0 (0%) |
| Services / sales (e.g., sales assistant, shelf filler, porter) | 1 (20%) | 1 (33%) | 0 (0%) |
| Catering (e.g., catering assistant, bar or hotel staff) | 1 (20%) | 0 (0%) | 1 (50%) |
| Other | 2 (40%) | 1 (33%) | 1 (50%) |
| **If yes, what was the salary level?** |  |  |  |
| Minimum wage | 3 (60%) | 2 (67%) | 1 (50%) |
| Other | 2 (40%) | 1 (33%) | 1 (50%) |
| **If yes, how many weeks during the 3 months was s/he employed in this occupation?** |  |  |  |
| 1 | 1 (20%) | 0 (0%) | 1 (50%) |
| 12 | 4 (80%) | 3 (100%) | 1 (50%) |
| **If yes, how many hours did s/he work per week?** |  |  |  |
| 1 -3 | 2 (40%) | 1 (33%) | 1 (50%) |
| 5 -8 | 2 (40%) | 1 (33%) | 1 (50%) |
| Over 20 | 1 (20%) | 1 (33%) | 0 (0%) |

Table S3: Baseline CSRI 4

|  | **Total (n = 36)** | **Intervention (n = 17)** | **Control (n = 19)** |
| --- | --- | --- | --- |
| **In the last 3 months, has the service user participated in any day activities?** |  |  |  |
| no | 14 (39%) | 8 (47%) | 6 (32%) |
| yes | 22 (61%) | 9 (53%) | 13 (68%) |
| **Day centre** |  |  |  |
| no | 15 (68%) | 8 (89%) | 7 (54%) |
| yes | 7 (32%) | 1 (11%) | 6 (46%) |
| **Provider sector** |  |  |  |
| Local authority | 2 (29%) | 0 (0%) | 2 (33%) |
| Voluntary organisation | 1 (14%) | 0 (0%) | 1 (17%) |
| Private | 4 (57%) | 1 (100%) | 3 (50%) |
| **How many weeks during the 3 months?** |  |  |  |
| 1 | 1 (14%) | 0 (0%) | 1 (17%) |
| 3 | 1 (14%) | 0 (0%) | 1 (17%) |
| 12 | 5 (71%) | 1 (100%) | 4 (67%) |
| **Total number of hours per week?** |  |  |  |
| 3 | 1 (14%) | 1 (100%) | 0 (0%) |
| 6 | 1 (14%) | 0 (0%) | 1 (17%) |
| 12 | 1 (14%) | 0 (0%) | 1 (17%) |
| 14 | 1 (14%) | 0 (0%) | 1 (17%) |
| 16 | 1 (14%) | 0 (0%) | 1 (17%) |
| 18 | 1 (14%) | 0 (0%) | 1 (17%) |
| 24 | 1 (14%) | 0 (0%) | 1 (17%) |
| **Sheltered work** |  |  |  |
| no | 22 (100%) | 9 (100%) | 13 (100%) |
| **Voluntary work** |  |  |  |
| no | 20 (91%) | 7 (78%) | 13 (100%) |
| yes | 2 (9%) | 2 (22%) | 0 (0%) |
| **Provider sector** |  |  |  |
| Local authority | 1 (50%) | 1 (50%) | 0 (0%) |
| Private | 1 (50%) | 1 (50%) | 0 (0%) |
| **How many weeks during the 3 months?** |  |  |  |
| 1 | 1 (50%) | 1 (50%) | 0 (0%) |
| 12 | 1 (50%) | 1 (50%) | 0 (0%) |
| **Total number of hours per week?** |  |  |  |
| 4 | 1 (50%) | 1 (50%) | 0 (0%) |
| 14 | 1 (50%) | 1 (50%) | 0 (0%) |
| **Adult education** |  |  |  |
| no | 17 (77%) | 8 (89%) | 9 (69%) |
| yes | 5 (23%) | 1 (11%) | 4 (31%) |
| **Provider sector** |  |  |  |
| Local authority | 3 (60%) | 1 (100%) | 2 (50%) |
| Private | 2 (40%) | 0 (0%) | 2 (50%) |
| **How many weeks during the 3 months?** |  |  |  |
| 1 | 1 (20%) | 0 (0%) | 1 (25%) |
| 2 | 1 (20%) | 0 (0%) | 1 (25%) |
| 12 | 3 (60%) | 1 (100%) | 2 (50%) |
| **Total number of hours per week?** |  |  |  |
| 4 | 2 (40%) | 0 (0%) | 2 (50%) |
| 7 | 1 (20%) | 0 (0%) | 1 (25%) |
| 18 | 1 (20%) | 1 (100%) | 0 (0%) |
| 22 | 1 (20%) | 0 (0%) | 1 (25%) |
| **Drop in centre** |  |  |  |
| no | 22 (100%) | 9 (100%) | 13 (100%) |
| **Social club** |  |  |  |
| no | 16 (73%) | 6 (67%) | 10 (77%) |
| yes | 6 (27%) | 3 (33%) | 3 (23%) |
| **Provider sector** |  |  |  |
| Voluntary organisation | 2 (33%) | 1 (33%) | 1 (33%) |
| Private | 4 (67%) | 2 (67%) | 2 (67%) |
| **How many weeks during the 3 months?** |  |  |  |
| 10 | 1 (17%) | 0 (0%) | 1 (33%) |
| 12 | 5 (83%) | 3 (100%) | 2 (67%) |
| **Total number of hours per week?** |  |  |  |
| 1 | 2 (40%) | 1 (33%) | 1 (50%) |
| 2 | 1 (20%) | 1 (33%) | 0 (0%) |
| 4 | 2 (40%) | 1 (33%) | 1 (50%) |
| **Recreation (e.g. swimming, horse riding)** |  |  |  |
| no | 13 (59%) | 3 (33%) | 10 (77%) |
| yes | 9 (41%) | 6 (67%) | 3 (23%) |
| **Provider sector** |  |  |  |
| Voluntary organisation | 2 (22%) | 1 (17%) | 1 (33%) |
| Private | 7 (78%) | 5 (83%) | 2 (67%) |
| **How many weeks during the 3 months?** |  |  |  |
| 5 | 1 (11%) | 0 (0%) | 1 (33%) |
| 12 | 8 (89%) | 6 (100%) | 2 (67%) |
| **Total number of hours per week?** |  |  |  |
| 1 | 1 (11%) | 0 (0%) | 1 (33%) |
| 2 | 1 (11%) | 1 (17%) | 0 (0%) |
| 3 | 2 (22%) | 1 (17%) | 1 (33%) |
| 4 | 1 (11%) | 1 (17%) | 0 (0%) |
| 5 | 2 (22%) | 2 (33%) | 0 (0%) |
| 8 | 1 (11%) | 0 (0%) | 1 (33%) |
| 20 | 1 (11%) | 1 (17%) | 0 (0%) |
| **Please specify recreation** |  |  |  |
| Sport (Gym, Rugby, swimming, walking etc) | 6 (16.7%) | 3 (17.6%) | 3 (15.7%) |
| Shopping, going out | 1 (2.8%) | 1 (17%) | 0 (0%) |
| **One-to-one activities** |  |  |  |
| no | 17 (77%) | 8 (89%) | 9 (69%) |
| yes | 5 (23%) | 1 (11%) | 4 (31%) |
| **Provider sector** |  |  |  |
| Private | 5 (100%) | 1 (100%) | 4 (100%) |
| **How many weeks during the 3 months?** |  |  |  |
| 1 | 1 (20%) | 1 (100%) | 0 (0%) |
| 12 | 4 (80%) | 0 (0%) | 4 (100%) |
| **Total number of hours per week?** |  |  |  |
| 3 | 1 (25%) | 0 (0%) | 1 (25%) |
| 5 | 1 (25%) | 0 (0%) | 1 (25%) |
| 10 | 1 (25%) | 0 (0%) | 1 (25%) |
| 14 | 1 (25%) | 0 (0%) | 1 (25%) |
| **Please specify one-to-one activities** |  |  |  |
| Bowling, cafe, shopping, bus rides | 1 (20%) | 0 (0%) | 1 (25%) |
| Holiday | 1 (20%) | 1 (100%) | 0 (0%) |
| Meals out, cinema, bowling, food shopping, visiting friends | 1 (20%) | 0 (0%) | 1 (25%) |
| Video games | 1 (20%) | 0 (0%) | 1 (25%) |
| Visiting places of interest e.g., museums, RAF bases | 1 (20%) | 0 (0%) | 1 (25%) |
| **Other activity** |  |  |  |
| no | 19 (86%) | 8 (89%) | 11 (85%) |
| yes | 3 (14%) | 1 (11%) | 2 (15%) |
| Provider sector |  |  |  |
| Private | 3 (100%) | 1 (100%) | 2 (100%) |
| **How many weeks during the 3 months?** |  |  |  |
| 6 | 1 (33%) | 1 (100%) | 0 (0%) |
| 12 | 2 (67%) | 0 (0%) | 2 (100%) |
| **Total number of hours per week?** |  |  |  |
| 1 | 1 (33%) | 0 (0%) | 1 (50%) |
| 2 | 1 (33%) | 1 (100%) | 0 (0%) |
| 3 | 1 (33%) | 0 (0%) | 1 (50%) |
| **Please specify other activity** |  |  |  |
| Bingo | 1 (33%) | 0 (0%) | 1 (50%) |
| Church | 2 (67%) | 1 (100%) | 1 (50%) |
| **How does the service user usually travel to day activities** |  |  |  |
| Transport provided by the residential facility | 6 (27%) | 2 (22%) | 4 (31%) |
| Personal transport | 4 (18%) | 4 (44%) | 0 (0%) |
| Family car | 3 (14%) | 0 (0%) | 3 (23%) |
| Public transport | 6 (27%) | 2 (22%) | 4 (31%) |
| Taxi | 1 (5%) | 0 (0%) | 1 (8%) |
| Other | 2 (9%) | 1 (11%) | 1 (8%) |
| **Other (please specify)** |  |  |  |
| Transport provided by support staff (own vehicles) from Q8 | 1 (50%) | 1 (100%) | 0 (0%) |
| Walk | 1 (50%) | 0 (0%) | 1 (100%) |
| **Does a member of care staff from the residential facility usually accompany the** |  |  |  |
| no | 4 (18%) | 1 (11%) | 3 (23%) |
| yes | 18 (82%) | 8 (89%) | 10 (77%) |

Table S4: Baseline CSRI 8-9

|  | **Total** | **Intervention (n = 17)** | **Control (n = 19)** |
| --- | --- | --- | --- |
| **Total – N (%)** | 36 (100.0%) | 17 (47.2%) | 19 (52.8%) |
| **Does the service user live in an unstaffed group home or supported living?** |  |  |  |
| No | 27 (75%) | 13 (76%) | 14 (74%) |
| Yes | 9 (25%) | 4 (24%) | 5 (26%) |
| **In the last 3 months, has the service user received additional paid support at home?** |  |  |  |
| No | 6 (75%) | 3 (75%) | 3 (75%) |
| Yes | 2 (25%) | 1 (25%) | 1 (25%) |
| **If yes, for how many weeks in the 3 months?** |  |  |  |
| 1 | 1 (50%) | 0 (0%) | 1 (100%) |
| 12 | 1 (50%) | 1 (100%) | 0 (0%) |
| **If yes, for how many hours per week?** |  |  |  |
| 40 | 1 (50%) | 1 (100%) | 0 (0%) |
| 48 | 1 (50%) | 0 (0%) | 1 (100%) |
| **If yes, was additional support made available because of the service-user’s anger/aggression?** |  |  |  |
| No | 2 (100%) | 1 (100%) | 1 (100%) |
| Yes | 0 (0%) | 0 (0%) | 0 (0%) |
| **Does the service user live in a staffed group home or supported living?** |  |  |  |
| No | 9 (25%) | 4 (24%) | 5 (26%) |
| Yes | 27 (75%) | 13 (76%) | 14 (74%) |
| **If yes, what is the total number of beds/places in the residential setting?** |  |  |  |
| 1 | 2 (7%) | 2 (15%) | 0 (0%) |
| 2 | 2 (7%) | 1 (8%) | 1 (7%) |
| 3 | 3 (11%) | 1 (8%) | 2 (14%) |
| 4 | 3 (11%) | 0 (0%) | 3 (21%) |
| 5 | 5 (19%) | 1 (8%) | 4 (29%) |
| 6 | 5 (19%) | 4 (31%) | 1 (7%) |
| 7 | 1 (4%) | 0 (0%) | 1 (7%) |
| 8 | 2 (7%) | 1 (8%) | 1 (7%) |
| 10 | 1 (4%) | 1 (8%) | 0 (0%) |
| 12 | 1 (4%) | 1 (8%) | 0 (0%) |
| 14 | 1 (4%) | 0 (0%) | 1 (7%) |
| 39 | 1 (4%) | 1 (8%) | 0 (0%) |
| **If yes, how many beds/places in the residential setting are currently occupied?** |  |  |  |
| 1 | 2 (7%) | 2 (15%) | 0 (0%) |
| 2 | 2 (7%) | 1 (8%) | 1 (7%) |
| 3 | 5 (19%) | 1 (8%) | 4 (29%) |
| 4 | 4 (15%) | 0 (0%) | 4 (29%) |
| 5 | 5 (19%) | 3 (23%) | 2 (14%) |
| 6 | 2 (7%) | 2 (15%) | 0 (0%) |
| 7 | 1 (4%) | 0 (0%) | 1 (7%) |
| 8 | 2 (7%) | 1 (8%) | 1 (7%) |
| 10 | 1 (4%) | 1 (8%) | 0 (0%) |
| 11 | 1 (4%) | 1 (8%) | 0 (0%) |
| 12 | 1 (4%) | 0 (0%) | 1 (7%) |
| 39 | 1 (4%) | 1 (8%) | 0 (0%) |
| **If yes, number of care staff usually on duty during the morning** |  |  |  |
| 1 | 5 (19%) | 2 (15%) | 3 (21%) |
| 2 | 6 (22%) | 2 (15%) | 4 (29%) |
| 3 | 6 (22%) | 3 (23%) | 3 (21%) |
| 4 | 1 (4%) | 0 (0%) | 1 (7%) |
| 5 | 6 (22%) | 4 (31%) | 2 (14%) |
| 7 | 1 (4%) | 0 (0%) | 1 (7%) |
| 12 | 1 (4%) | 1 (8%) | 0 (0%) |
| 22 | 1 (4%) | 1 (8%) | 0 (0%) |
| **If yes, number of staff usually on duty during the afternoon** |  |  |  |
| 1 | 5 (19%) | 2 (15%) | 3 (21%) |
| 2 | 7 (26%) | 3 (23%) | 4 (29%) |
| 3 | 6 (22%) | 2 (15%) | 4 (29%) |
| 4 | 1 (4%) | 0 (0%) | 1 (7%) |
| 5 | 5 (19%) | 4 (31%) | 1 (7%) |
| 7 | 1 (4%) | 0 (0%) | 1 (7%) |
| 8 | 1 (4%) | 1 (8%) | 0 (0%) |
| 12 | 1 (4%) | 1 (8%) | 0 (0%) |
| **If yes, number of staff usually on duty during the evening** |  |  |  |
| 1 | 4 (15%) | 1 (8%) | 3 (21%) |
| 2 | 9 (33%) | 4 (31%) | 5 (36%) |
| 3 | 5 (19%) | 2 (15%) | 3 (21%) |
| 4 | 3 (11%) | 2 (15%) | 1 (7%) |
| 5 | 3 (11%) | 2 (15%) | 1 (7%) |
| 6 | 1 (4%) | 0 (0%) | 1 (7%) |
| 12 | 1 (4%) | 1 (8%) | 0 (0%) |
| 18 | 1 (4%) | 1 (8%) | 0 (0%) |
| **If yes, number of staff usually on duty at night** |  |  |  |
| 0 | 2 (7%) | 0 (0%) | 2 (14%) |
| 1 | 16 (59%) | 7 (54%) | 9 (64%) |
| 2 | 5 (19%) | 3 (23%) | 2 (14%) |
| 3 | 1 (4%) | 1 (8%) | 0 (0%) |
| 4 | 2 (7%) | 1 (8%) | 1 (7%) |
| 6 | 1 (4%) | 1 (8%) | 0 (0%) |

Table S5: Baseline DIBELS

|  | **Total (n = 36)** | **Intervention (n = 17)** | **Control (n = 19)** |
| --- | --- | --- | --- |
| **Letter naming fluency (LNF), median (IQR)** | 17.0 (7.0, 23.0) | 18.0 (8.0, 23.0) | 16.0 (6.0, 23.0) |
| **Phonemic segmentation fluency (PSF), median (IQR)** | 1.0 (0.0, 2.0) | 0.0 (0.0, 1.0) | 2.0 (0.0, 3.0) |
| **Correct letter sounds (CLS), median (IQR)** | 3.0 (1.0, 7.5) | 4.0 (2.0, 5.0) | 3.0 (1.0, 13.0) |
| **Words read correctly (WRC), median (IQR)** | 0.0 (0.0, 0.0) | 0.0 (0.0, 0.0) | 0.0 (0.0, 1.0) |
| **Word reading fluency (WRF), median (IQR)** | 2.0 (1.0, 7.5) | 2.0 (1.0, 8.0) | 2.0 (1.0, 5.0) |
| **Words Correct, median (IQR)** | 4.0 (2.0, 14.0) | 5.0 (2.0, 16.0) | 4.0 (3.0, 9.0) |
| **Errors, median (IQR)** | 8.0 (6.0, 12.0) | 8.0 (6.0, 12.0) | 8.0 (6.0, 12.0) |
| **Accuracy, median (IQR)** | 0.4 (0.2, 0.7) | 0.5 (0.1, 0.7) | 0.3 (0.2, 0.7) |
| **Composite score** | 349.0 (344.9, 354.4) | 352.2 (246.2, 254.4) | 347.7 (344.8, 351.0) |

Table S6: Baseline EQ5D-3L

|  | **Total (n = 36)** | **Intervention (n = 17)** | **Control (n = 19)** |
| --- | --- | --- | --- |
| **Mobility** |  |  |  |
| I have no problems in walking about | 28 (78%) | 14 (82%) | 14 (74%) |
| I have some problems in walking about | 8 (22%) | 3 (18%) | 5 (26%) |
| I am confined to bed | 0 (0%) | 0 (0%) | 0 (0%) |
| **Self-care** |  |  |  |
| I have no problems with self-care | 28 (78%) | 13 (76%) | 15 (79%) |
| I have some problems washing or dressing myself | 8 (22%) | 4 (24%) | 4 (21%) |
| I am unable to wash or dress myself | 0 (0%) | 0 (0%) | 0 (0%) |
| **Usual Activities** |  |  |  |
| I have no problems with performing my usual activities | 28 (78%) | 12 (71%) | 16 (84%) |
| I have some problems with performing my usual activities | 8 (22%) | 5 (29%) | 3 (16%) |
| I am unable to perform my usual activities | 0 (0%) | 0 (0%) | 0 (0%) |
| **Pain / Discomfort** |  |  |  |
| I have no pain or discomfort | 32 (89%) | 15 (88%) | 17 (89%) |
| I have moderate pain or discomfort | 3 (8%) | 1 (6%) | 2 (11%) |
| I have extreme pain or discomfort | 1 (3%) | 1 (6%) | 0 (0%) |
| **Anxiety / Depression** |  |  |  |
| I am not anxious or depressed | 25 (69%) | 15 (88%) | 10 (53%) |
| I am moderately anxious or depressed | 10 (28%) | 2 (12%) | 8 (42%) |
| I am extremely anxious or depressed | 1 (3%) | 0 (0%) | 1 (5%) |
| **Your health today, median (IQR)** | 90.0 (75.5, 100.0) | 90.0 (76.0, 100.0) | 90.0 (75.0, 100.0) |
| **Final EQ5D Index, median (IQR)** | 0.9 (0.8, 1.0) | 0.9 (0.8, 1.0) | 0.8 (0.7, 1.0) |

Table S7: Baseline PWI

|  | **Total (n = 36)** | **Intervention (n = 17)** | **Control (n = 19)** |
| --- | --- | --- | --- |
| **How happy do you feel about your life as a whole?** |  |  |  |
| Not at all happy | 0 (0%) | 0 (0%) | 0 (0%) |
| A little bit happy | 2 (6%) | 0 (0%) | 2 (11%) |
| Happy | 19 (53%) | 10 (59%) | 9 (47%) |
| Very Happy | 15 (42%) | 7 (41%) | 8 (42%) |
| **How happy do you feel about the things you have?** |  |  |  |
| Not at all happy | 2 (6%) | 0 (0%) | 2 (11%) |
| A little bit happy | 0 (0%) | 0 (0%) | 0 (0%) |
| Happy | 11 (31%) | 4 (24%) | 7 (37%) |
| Very Happy | 23 (64%) | 13 (76%) | 10 (53%) |
| **How happy do you feel about how healthy you are?** |  |  |  |
| Not at all happy | 2 (6%) | 2 (12%) | 0 (0%) |
| A little bit happy | 7 (19%) | 1 (6%) | 6 (32%) |
| Happy | 14 (39%) | 8 (47%) | 6 (32%) |
| Very Happy | 13 (36%) | 6 (35%) | 7 (37%) |
| **How happy do you feel about the things you make or the things you learn?** |  |  |  |
| Not at all happy | 0 (0%) | 0 (0%) | 0 (0%) |
| A little bit happy | 3 (8%) | 0 (0%) | 3 (16%) |
| Happy | 14 (39%) | 6 (35%) | 8 (42%) |
| Very Happy | 19 (53%) | 11 (65%) | 8 (42%) |
| **How happy do you feel about getting on with the people you know?** |  |  |  |
| Not at all happy | 0 (0%) | 0 (0%) | 0 (0%) |
| A little bit happy | 4 (11%) | 0 (0%) | 4 (21%) |
| Happy | 11 (31%) | 3 (18%) | 8 (42%) |
| Very Happy | 21 (58%) | 14 (82%) | 7 (37%) |
| **How happy do you feel about how safe you feel?** |  |  |  |
| Not at all happy | 0 (0%) | 0 (0%) | 0 (0%) |
| A little bit happy | 5 (14%) | 2 (12%) | 3 (16%) |
| Happy | 14 (39%) | 5 (29%) | 9 (47%) |
| Very Happy | 17 (47%) | 10 (59%) | 7 (37%) |
| **How happy do you feel about doing things outside your home?** |  |  |  |
| Not at all happy | 1 (3%) | 0 (0%) | 1 (5%) |
| A little bit happy | 5 (14%) | 1 (6%) | 4 (21%) |
| Happy | 10 (28%) | 5 (29%) | 5 (26%) |
| Very Happy | 20 (56%) | 11 (65%) | 9 (47%) |
| **How happy do you feel about how things will be later on in your life?** |  |  |  |
| Not at all happy | 1 (3%) | 1 (6%) | 0 (0%) |
| A little bit happy | 5 (14%) | 1 (6%) | 4 (21%) |
| Happy | 19 (53%) | 9 (53%) | 10 (53%) |
| Very Happy | 11 (31%) | 6 (35%) | 5 (26%) |
| **Raw score, median (IQR)** | 27.0 (25.0, 29.0) | 28.0 (27.0, 29.0) | 25.0 (23.0, 28.0) |
| **Final score, median (IQR)** | 79.2 (70.8, 87.5) | 83.3 (79.2, 87.5) | 70.8 (62.5, 83.3) |

Table S8: Baseline RSCS

|  | **Total (n = 36)** | **Intervention (n = 17)** | **Control (n = 19)** |
| --- | --- | --- | --- |
| **I need help to read things like instructions, recipes, packages on food, letters** |  |  |  |
| Never | 1 (3%) | 0 (0%) | 1 (5%) |
| Sometimes | 10 (28%) | 3 (18%) | 7 (37%) |
| Always | 25 (69%) | 14 (82%) | 11 (58%) |
| **Needing help to read makes me feel inadequate** |  |  |  |
| Never | 12 (33%) | 6 (35%) | 6 (32%) |
| Sometimes | 17 (47%) | 8 (47%) | 9 (47%) |
| Always | 7 (19%) | 3 (18%) | 4 (21%) |
| **Reading is easy for me** |  |  |  |
| Never | 18 (50%) | 10 (59%) | 8 (42%) |
| Sometimes | 15 (42%) | 5 (29%) | 10 (53%) |
| Always | 3 (8%) | 2 (12%) | 1 (5%) |
| **Reading is important to me** |  |  |  |
| Never | 2 (6%) | 2 (12%) | 0 (0%) |
| Sometimes | 19 (53%) | 5 (29%) | 14 (74%) |
| Always | 15 (42%) | 10 (59%) | 5 (26%) |
| **I get words wrong when I read** |  |  |  |
| Never | 5 (14%) | 2 (12%) | 3 (16%) |
| Sometimes | 18 (50%) | 10 (59%) | 8 (42%) |
| Always | 13 (36%) | 5 (29%) | 8 (42%) |
| **Reading helps me keep in touch with people** |  |  |  |
| Never | 5 (14%) | 2 (12%) | 3 (16%) |
| Sometimes | 6 (17%) | 0 (0%) | 6 (32%) |
| Always | 25 (69%) | 15 (88%) | 10 (53%) |

Table S9: Baseline SRS

|  | **Total (n = 36)** | **Intervention (n = 17)** | **Control (n = 19)** |
| --- | --- | --- | --- |
| **How confident are you in helping the person you support to read?** |  |  |  |
| 1 - Not at all confident | 0 (0%) | 0 (0%) | 0 (0%) |
| 2 | 0 (0%) | 0 (0%) | 0 (0%) |
| 3 | 0 (0%) | 0 (0%) | 0 (0%) |
| 4 | 2 (6%) | 1 (6%) | 1 (5%) |
| 5 | 4 (11%) | 1 (6%) | 3 (16%) |
| 6 | 9 (25%) | 4 (24%) | 5 (26%) |
| 7 - Very confident | 21 (58%) | 11 (65%) | 10 (53%) |
| **How difficult do you find it to help the person you support to understand the things they need to read?** |  |  |  |
| 1 - Very difficult | 2 (6%) | 2 (12%) | 0 (0%) |
| 2 | 1 (3%) | 1 (6%) | 0 (0%) |
| 3 | 1 (3%) | 0 (0%) | 1 (5%) |
| 4 | 5 (14%) | 4 (24%) | 1 (5%) |
| 5 | 9 (25%) | 4 (24%) | 5 (26%) |
| 6 | 7 (19%) | 0 (0%) | 7 (37%) |
| 7 - Not at all difficult | 11 (31%) | 6 (35%) | 5 (26%) |
| **To what extent do you feel that the things that you do to help the person you support to read have a positive effect?** |  |  |  |
| 1 – Have a very negative effect | 0 (0%) | 0 (0%) | 0 (0%) |
| 2 | 0 (0%) | 0 (0%) | 0 (0%) |
| 3 | 2 (6%) | 1 (6%) | 1 (5%) |
| 4 | 2 (6%) | 1 (6%) | 1 (5%) |
| 5 | 5 (14%) | 2 (12%) | 3 (16%) |
| 6 | 13 (36%) | 5 (29%) | 8 (42%) |
| 7 - Have a very positive effect | 14 (39%) | 8 (47%) | 6 (32%) |
| **How satisfied are you with the way in which you help the person you support to understand the things they need to read?** |  |  |  |
| 1 - Not satisfied at all | 1 (3%) | 1 (6%) | 0 (0%) |
| 2 | 1 (3%) | 1 (6%) | 0 (0%) |
| 3 | 3 (8%) | 2 (12%) | 1 (5%) |
| 4 | 3 (8%) | 2 (12%) | 1 (5%) |
| 5 | 8 (22%) | 3 (18%) | 5 (26%) |
| 6 | 12 (33%) | 5 (29%) | 7 (37%) |
| 7 - Very satisfied | 8 (22%) | 3 (18%) | 5 (26%) |

Table S10: Follow-up CSRI 1-3

|  | **Total (n = 31)** | **Intervention (n = 17)** | **Control (n = 14)** |
| --- | --- | --- | --- |
| **Total – N (%)** |  |  |  |
| **What is the job title of the support worker?** |  |  |  |
| Support worker/Carer | 14 (45,2%) | 6 (35.3%) | 8 (5.7%) |
| Team/Autism lead | 5 (16,1%) | 4 (23.5%) | 1 (7.1%) |
| Registered/deputy/Care manager | 5 (16.1%) | 2 (11.7%) | 3 (21.4%) |
| Retired | 1 (3%) | 1 (5.9%) | 0 (0%) |
| Senior social care worker | 1 (3%) | 1 (5.9%) | 0 (0%) |
| Family carer Sister | 3 (9.7%) | 1 (5.9%) | 2 (14.2%) |
| **What is the service user’s usual place of residence?** |  |  |  |
| Staffed group home or supported living | 21 (72%) | 12 (80%) | 9 (64%) |
| Family home | 6 (21%) | 2 (13%) | 4 (29%) |
| Adult family placement | 2 (7%) | 1 (7%) | 1 (7%) |
| **In the last 3 months, has the service user done any paid work?** |  |  |  |
| no | 27 (93%) | 13 (87%) | 14 (100%) |
| yes | 2 (7%) | 2 (13%) | 0 (0%) |
| **If yes, what was/is the service users occupation?** |  |  |  |
| Services / sales (e.g., sales assistant, shelf filler, porter) | 1 (50%) | 1 (50%) | 0 (0%) |
| Other | 1 (50%) | 1 (50%) | 0 (0%) |
| **If yes, what was the salary level?** |  |  |  |
| Minimum wage | 2 (100%) | 2 (100%) | 0 (0%) |
| **If yes, how many weeks during the 3 months was s/he employed in this occupation?** |  |  |  |
| 12 | 2 (100%) | 2 (100%) | 0 (0%) |
| **If yes, how many hours did s/he work per week?** |  |  |  |
| 8 | 1 (50%) | 1 (50%) | 0 (0%) |
| 15 | 1 (50%) | 1 (50%) | 0 (0%) |

Table S11: Follow-up CSRI 4

|  | **Total (n = 31)** | **Intervention (n = 17)** | **Control (n = 14)** |
| --- | --- | --- | --- |
| **In the last 3 months, has the service user participated in any day activities?** |  |  |  |
| no | 8 (28%) | 6 (40%) | 2 (14%) |
| yes | 21 (72%) | 9 (60%) | 12 (86%) |
| **Day centre** |  |  |  |
| no | 10 (48%) | 5 (56%) | 5 (42%) |
| yes | 11 (52%) | 4 (44%) | 7 (58%) |
| **Provider sector** |  |  |  |
| Local authority | 3 (27%) | 1 (25%) | 2 (29%) |
| Voluntary organisation | 2 (18%) | 0 (0%) | 2 (29%) |
| Private | 6 (55%) | 3 (75%) | 3 (43%) |
| **How many weeks during the 3 months?** |  |  |  |
| 1 | 1 (9%) | 1 (25%) | 0 (0%) |
| 4 | 1 (9%) | 0 (0%) | 1 (14%) |
| 5 | 1 (9%) | 0 (0%) | 1 (14%) |
| 7 | 1 (9%) | 0 (0%) | 1 (14%) |
| 8 | 1 (9%) | 1 (25%) | 0 (0%) |
| 10 | 1 (9%) | 0 (0%) | 1 (14%) |
| 12 | 5 (45%) | 2 (50%) | 3 (43%) |
| **Total number of hours per week?** |  |  |  |
| 2 | 2 (18%) | 1 (25%) | 1 (14%) |
| 5 | 2 (18%) | 1 (25%) | 1 (14%) |
| 6 | 1 (9%) | 1 (25%) | 0 (0%) |
| 10 | 1 (9%) | 0 (0%) | 1 (14%) |
| 12 | 1 (9%) | 0 (0%) | 1 (14%) |
| 14 | 1 (9%) | 0 (0%) | 1 (14%) |
| 15 | 1 (9%) | 1 (25%) | 0 (0%) |
| 18 | 1 (9%) | 0 (0%) | 1 (14%) |
| 35 | 1 (9%) | 0 (0%) | 1 (14%) |
| **Sheltered work** |  |  |  |
| no | 21 (100%) | 9 (100%) | 12 (100%) |
| **Voluntary work** |  |  |  |
| no | 15 (71%) | 6 (67%) | 9 (75%) |
| yes | 6 (29%) | 3 (33%) | 3 (25%) |
| **Provider sector** |  |  |  |
| Voluntary organisation | 2 (33%) | 0 (0%) | 2 (67%) |
| Private | 4 (67%) | 3 (100%) | 1 (33%) |
| **How many weeks during the 3 months?** |  |  |  |
| 5 | 1 (17%) | 0 (0%) | 1 (33%) |
| 6 | 1 (17%) | 0 (0%) | 1 (33%) |
| 12 | 4 (67%) | 3 (100%) | 1 (33%) |
| **Total number of hours per week?** |  |  |  |
| 2 | 4 (67%) | 2 (67%) | 2 (67%) |
| 4 | 1 (17%) | 0 (0%) | 1 (33%) |
| 12 | 1 (17%) | 1 (33%) | 0 (0%) |
| **Adult education** |  |  |  |
| no | 16 (76%) | 7 (78%) | 9 (75%) |
| yes | 5 (24%) | 2 (22%) | 3 (25%) |
| **Provider sector** |  |  |  |
| Local authority | 4 (80%) | 2 (100%) | 2 (67%) |
| Private | 1 (20%) | 0 (0%) | 1 (33%) |
| **How many weeks during the 3 months?** |  |  |  |
| 3 | 1 (20%) | 1 (50%) | 0 (0%) |
| 8 | 3 (60%) | 1 (50%) | 2 (67%) |
| 12 | 1 (20%) | 0 (0%) | 1 (33%) |
| **Total number of hours per week?** |  |  |  |
| 2 | 1 (20%) | 1 (50%) | 0 (0%) |
| 4 | 1 (20%) | 1 (50%) | 0 (0%) |
| 12 | 2 (40%) | 0 (0%) | 2 (67%) |
| 20 | 1 (20%) | 0 (0%) | 1 (33%) |
| **Drop in centre** |  |  |  |
| no | 21 (100%) | 9 (100%) | 12 (100%) |
| **Social club** |  |  |  |
| no | 12 (57%) | 4 (44%) | 8 (67%) |
| yes | 9 (43%) | 5 (56%) | 4 (33%) |
| **Provider sector** |  |  |  |
| Voluntary organisation | 4 (44%) | 2 (40%) | 2 (50%) |
| Private | 5 (56%) | 3 (60%) | 2 (50%) |
| **How many weeks during the 3 months?** |  |  |  |
| 3 | 2 (22%) | 0 (0%) | 2 (50%) |
| 6 | 2 (22%) | 2 (40%) | 0 (0%) |
| 9 | 1 (11%) | 0 (0%) | 1 (25%) |
| 12 | 4 (44%) | 3 (60%) | 1 (25%) |
| **Total number of hours per week?** |  |  |  |
| 2 | 7 (78%) | 5 (100%) | 2 (50%) |
| 3 | 2 (22%) | 0 (0%) | 2 (50%) |
| **Recreation (e.g. swimming, horse riding)** |  |  |  |
| no | 20 (95%) | 9 (100%) | 11 (92%) |
| yes | 1 (5%) | 0 (0%) | 1 (8%) |
| **Provider sector** |  |  |  |
| Voluntary organisation | 1 (100%) | 0 (0%) | 1 (100%) |
| **How many weeks during the 3 months?** |  |  |  |
| 12 | 1 (100%) | 0 (0%) | 1 (100%) |
| **Total number of hours per week?** |  |  |  |
| 2 | 1 (100%) | 0 (0%) | 1 (100%) |
| **Please specify recreation** |  |  |  |
| Rugby | 1 (100%) | 0 (0%) | 1 (100%) |
| **One-to-one activities** |  |  |  |
| no | 21 (100%) | 9 (100%) | 12 (100%) |
| **How many weeks during the 3 months?** |  |  |  |
| 12 | 1 (100%) | 0 (0%) | 1 (100%) |
| **Total number of hours per week?** |  |  |  |
| 2 | 1 (100%) | 0 (0%) | 1 (100%) |
| **Please specify one-to-one activities** |  |  |  |
| Church | 1 (33%) | 0 (0%) | 1 (100%) |
| **How does the service user usually travel to day activities** |  |  |  |
| Transport provided by the residential facility | 4 (19%) | 2 (22%) | 2 (17%) |
| Transport provided by the day activity organisation | 1 (5%) | 0 (0%) | 1 (8%) |
| Personal transport | 2 (10%) | 2 (22%) | 0 (0%) |
| Family car | 4 (19%) | 0 (0%) | 4 (33%) |
| Public transport | 5 (24%) | 4 (44%) | 1 (8%) |
| Taxi | 1 (5%) | 0 (0%) | 1 (8%) |
| Other | 4 (19%) | 1 (11%) | 3 (25%) |
| **Other (please specify)** |  |  |  |
| Friend's car | 1 (25%) | 1 (100%) | 0 (0%) |
| Personal car of another adult with LD who live with | 1 (25%) | 0 (0%) | 1 (33%) |
| Walk | 1 (25%) | 0 (0%) | 1 (33%) |
| Walking | 1 (25%) | 0 (0%) | 1 (33%) |
| **Does a member of care staff from the residential facility usually accompany the** |  |  |  |
| no | 5 (24%) | 2 (22%) | 3 (25%) |
| yes | 16 (76%) | 7 (78%) | 9 (75%) |

Table S12: Follow-up CSRI 8-9

|  | **Total (n = 31)** | **Intervention (n = 17)** | **Control (n = 14)** |
| --- | --- | --- | --- |
| **Total – N (%)** |  |  |  |
| **Does the service user live in an unstaffed group home or supported living?** |  |  |  |
| No | 21 (72%) | 12 (80%) | 9 (64%) |
| Yes | 8 (28%) | 3 (20%) | 5 (36%) |
| **In the last 3 months, has the service user received additional paid support at home?** |  |  |  |
| No | 6 (86%) | 2 (67%) | 4 (100%) |
| Yes | 1 (14%) | 1 (33%) | 0 (0%) |
| **If yes, for how many weeks in the 3 months?** |  |  |  |
| 12 | 1 (100%) | 1 (100%) | 0 (0%) |
| **If yes, for how many hours per week?** |  |  |  |
| 40 | 1 (100%) | 1 (100%) | 0 (0%) |
| **If yes, was additional support made available because of the service-user’s anger/aggression?** |  |  |  |
| No | 1 (100%) | 1 (100%) | 0 (0%) |
| **Does the service user live in a staffed group home or supported living?** |  |  |  |
| Yes | 21 (100%) | 12 (100%) | 9 (100%) |
| **If yes, what is the total number of beds/places in the residential setting?** |  |  |  |
| 1 | 2 (10%) | 2 (17%) | 0 (0%) |
| 3 | 4 (19%) | 2 (17%) | 2 (22%) |
| 4 | 1 (5%) | 0 (0%) | 1 (11%) |
| 5 | 4 (19%) | 1 (8%) | 3 (33%) |
| 6 | 4 (19%) | 4 (33%) | 0 (0%) |
| 8 | 2 (10%) | 0 (0%) | 2 (22%) |
| 10 | 1 (5%) | 1 (8%) | 0 (0%) |
| 12 | 1 (5%) | 1 (8%) | 0 (0%) |
| 14 | 1 (5%) | 0 (0%) | 1 (11%) |
| 39 | 1 (5%) | 1 (8%) | 0 (0%) |
| **If yes, how many beds/places in the residential setting are currently occupied?** |  |  |  |
| 1 | 2 (10%) | 2 (17%) | 0 (0%) |
| 2 | 1 (5%) | 1 (8%) | 0 (0%) |
| 3 | 4 (19%) | 1 (8%) | 3 (33%) |
| 4 | 2 (10%) | 0 (0%) | 2 (22%) |
| 5 | 4 (19%) | 2 (17%) | 2 (22%) |
| 6 | 3 (14%) | 3 (25%) | 0 (0%) |
| 8 | 1 (5%) | 0 (0%) | 1 (11%) |
| 10 | 1 (5%) | 1 (8%) | 0 (0%) |
| 11 | 1 (5%) | 1 (8%) | 0 (0%) |
| 13 | 1 (5%) | 0 (0%) | 1 (11%) |
| 38 | 1 (5%) | 1 (8%) | 0 (0%) |
| **If yes, number of care staff usually on duty during the morning** |  |  |  |
| 1 | 4 (19%) | 3 (25%) | 1 (11%) |
| 2 | 4 (19%) | 1 (8%) | 3 (33%) |
| 3 | 4 (19%) | 2 (17%) | 2 (22%) |
| 4 | 1 (5%) | 1 (8%) | 0 (0%) |
| 5 | 6 (29%) | 3 (25%) | 3 (33%) |
| 12 | 1 (5%) | 1 (8%) | 0 (0%) |
| 15 | 1 (5%) | 1 (8%) | 0 (0%) |
| **If yes, number of staff usually on duty during the afternoon** |  |  |  |
| 1 | 4 (19%) | 3 (25%) | 1 (11%) |
| 2 | 4 (19%) | 1 (8%) | 3 (33%) |
| 3 | 4 (19%) | 2 (17%) | 2 (22%) |
| 5 | 7 (33%) | 4 (33%) | 3 (33%) |
| 12 | 1 (5%) | 1 (8%) | 0 (0%) |
| 15 | 1 (5%) | 1 (8%) | 0 (0%) |
| **If yes, number of staff usually on duty during the evening** |  |  |  |
| 1 | 4 (19%) | 3 (25%) | 1 (11%) |
| 2 | 5 (24%) | 1 (8%) | 4 (44%) |
| 3 | 4 (19%) | 3 (25%) | 1 (11%) |
| 5 | 6 (29%) | 3 (25%) | 3 (33%) |
| 12 | 2 (10%) | 2 (17%) | 0 (0%) |
| **If yes, number of staff usually on duty at night** |  |  |  |
| 0 | 1 (5%) | 0 (0%) | 1 (11%) |
| 1 | 12 (57%) | 6 (50%) | 6 (67%) |
| 2 | 4 (19%) | 4 (33%) | 0 (0%) |
| 3 | 2 (10%) | 1 (8%) | 1 (11%) |
| 4 | 2 (10%) | 1 (8%) | 1 (11%) |
| **Did the service user receive any reading support or teaching in the previous 6m?** |  |  |  |
| No | 13 (93%) | 1 (100%) | 12 (92%) |
| Yes | 1 (7%) | 0 (0%) | 1* (8%) |

*Comment from participant “*Some small bits of reading as part of adult education courses – not main focus of these – very small*”

Table S13: Follow-up DIBELS

|  | **Total (n = 31)** | **Intervention (n = 17)** | **Control (n = 14)** |
| --- | --- | --- | --- |
| **Letter naming fluency (LNF), median (IQR)** | 18.0 (8.0, 27.0) | 18.0 (10.0, 27.0) | 17.5 (8.0, 30.0) |
| **Phonemic segmentation fluency (PSF), median (IQR)** | 1.0 (0.0, 4.0) | 1.0 (0.0, 3.0) | 0.0 (0.0, 6.0) |
| **Correct letter sounds (CLS), median (IQR)** | 3.0 (1.0, 17.0) | 8.0 (0.0, 17.0) | 2.5 (1.0, 27.0) |
| **Words read correctly (WRC), median (IQR)** | 0.0 (0.0, 1.0) | 0.0 (0.0, 0.0) | 0.0 (0.0, 7.0) |
| **Word reading fluency (WRF), median (IQR)** | 3.0 (0.0, 11.0) | 2.0 (0.0, 6.0) | 5.5 (1.0, 12.0) |
| **Words Correct, median (IQR)** | 1.0 (0.0, 9.0) | 1.0 (0.0, 5.0) | 3.5 (0.0, 33.0) |
| **Errors, median (IQR)** | 9.0 (8.0, 10.0) | 9.0 (7.0, 11.0) | 9.0 (9.0, 10.0) |
| **Accuracy, median (IQR)** | 0.1 (0.0, 0.6) | 0.1 (0.0, 0.6) | 0.3 (0.0, 0.8) |
| **Composite score** | 347.8 (345.0, 354.4) | 351.8 (344.2, 354.4) | 346.8 (345.0, 361.7) |

Table S14: Follow-up EQ5D-3L

|  | **Total (n = 31)** | **Intervention (n = 17)** | **Control (n = 14)** |
| --- | --- | --- | --- |
| **Mobility** |  |  |  |
| I have no problems in walking about | 23 (79%) | 12 (80%) | 11 (79%) |
| I have some problems in walking about | 6 (21%) | 3 (20%) | 3 (21%) |
| I am confined to bed | 0 (0%) | 0 (0%) | 0 (0%) |
| **Self-care** |  |  |  |
| I have no problems with self-care | 23 (79%) | 13 (87%) | 10 (71%) |
| I have some problems washing or dressing myself | 6 (21%) | 2 (13%) | 4 (29%) |
| I am unable to wash or dress myself | 0 (0%) | 0 (0%) | 0 (0%) |
| **Usual Activities** |  |  |  |
| I have no problems with performing my usual activities | 28 (97%) | 15 (100%) | 13 (93%) |
| I have some problems with performing my usual activities | 1 (3%) | 0 (0%) | 1 (7%) |
| I am unable to perform my usual activities | 0 (0%) | 0 (0%) | 0 (0%) |
| **Pain / Discomfort** |  |  |  |
| I have no pain or discomfort | 27 (93%) | 15 (100%) | 12 (86%) |
| I have moderate pain or discomfort | 2 (7%) | 0 (0%) | 2 (14%) |
| I have extreme pain or discomfort | 0 (0%) | 0 (0%) | 0 (0%) |
| **Anxiety / Depression** |  |  |  |
| I am not anxious or depressed | 24 (83%) | 13 (87%) | 11 (79%) |
| I am moderately anxious or depressed | 4 (14%) | 1 (7%) | 3 (21%) |
| I am extremely anxious or depressed | 1 (3%) | 1 (7%) | 0 (0%) |
| **Your health today, median (IQR)** | 100.0 (65.0, 100.0) | 100.0 (60.0, 100.0) | 90.0 (65.0, 100.0) |
| **Final EQ5D Index, median (IQR)** | 1.0 (0.7, 1.0) | 1.0 (0.8, 1.0) | 1.0 (0.7, 1.0) |

Table S15: Follow-up PWI

|  | **Total (n = 31)** | **Intervention (n = 17)** | **Control (n = 14)** |
| --- | --- | --- | --- |
| **How happy do you feel about your life as a whole?** |  |  |  |
| Not at all happy | 0 (0%) | 0 (0%) | 0 (0%) |
| A little bit happy | 1 (3%) | 0 (0%) | 1 (7%) |
| Happy | 14 (48%) | 8 (53%) | 6 (43%) |
| Very Happy | 14 (48%) | 7 (47%) | 7 (50%) |
| **How happy do you feel about the things you have?** |  |  |  |
| Not at all happy | 0 (0%) | 0 (0%) | 0 (0%) |
| A little bit happy | 2 (7%) | 0 (0%) | 2 (14%) |
| Happy | 13 (45%) | 5 (33%) | 8 (57%) |
| Very Happy | 14 (48%) | 10 (67%) | 4 (29%) |
| **How happy do you feel about how healthy you are?** |  |  |  |
| Not at all happy | 0 (0%) | 0 (0%) | 0 (0%) |
| A little bit happy | 2 (7%) | 1 (7%) | 1 (7%) |
| Happy | 14 (48%) | 5 (33%) | 9 (64%) |
| Very Happy | 13 (45%) | 9 (60%) | 4 (29%) |
| **How happy do you feel about the things you make or the things you learn?** |  |  |  |
| Not at all happy | 1 (3%) | 0 (0%) | 1 (7%) |
| A little bit happy | 2 (7%) | 0 (0%) | 2 (14%) |
| Happy | 11 (38%) | 6 (40%) | 5 (36%) |
| Very Happy | 15 (52%) | 9 (60%) | 6 (43%) |
| **How happy do you feel about getting on with the people you know?** |  |  |  |
| Not at all happy | 0 (0%) | 0 (0%) | 0 (0%) |
| A little bit happy | 2 (7%) | 1 (7%) | 1 (7%) |
| Happy | 12 (41%) | 4 (27%) | 8 (57%) |
| Very Happy | 15 (52%) | 10 (67%) | 5 (36%) |
| **How happy do you feel about how safe you feel?** |  |  |  |
| Not at all happy | 0 (0%) | 0 (0%) | 0 (0%) |
| A little bit happy | 6 (21%) | 1 (7%) | 5 (36%) |
| Happy | 11 (38%) | 6 (40%) | 5 (36%) |
| Very Happy | 12 (41%) | 8 (53%) | 4 (29%) |
| **How happy do you feel about doing things outside your home?** |  |  |  |
| Not at all happy | 0 (0%) | 0 (0%) | 0 (0%) |
| A little bit happy | 2 (7%) | 0 (0%) | 2 (14%) |
| Happy | 12 (41%) | 4 (27%) | 8 (57%) |
| Very Happy | 15 (52%) | 11 (73%) | 4 (29%) |
| **How happy do you feel about how things will be later on in your life?** |  |  |  |
| Not at all happy | 0 (0%) | 0 (0%) | 0 (0%) |
| A little bit happy | 3 (13%) | 1 (8%) | 2 (18%) |
| Happy | 10 (42%) | 5 (38%) | 5 (45%) |
| Very Happy | 11 (46%) | 7 (54%) | 4 (36%) |
| **Raw score, median (IQR)** | 28.0 (24.5, 30.0) | 29.0 (26.0, 31.0) | 25.0 (23.0, 29.0) |
| **Final score, median (IQR)** | 83.3 (68.8, 91.7) | 87.5 (75.0, 95.8) | 70.8 (62.5, 87.5) |

Table S16: Follow-up RSCS

|  | **Total (n = 31)** | **Intervention (n = 17)** | **Control (n = 14)** |
| --- | --- | --- | --- |
| **I need help to read things like instructions, recipes, packages on food, letters** |  |  |  |
| Never | 0 (0%) | 0 (0%) | 0 (0%) |
| Sometimes | 8 (28%) | 2 (13%) | 6 (43%) |
| Always | 21 (72%) | 13 (87%) | 8 (57%) |
| **Needing help to read makes me feel inadequate** |  |  |  |
| Never | 11 (39%) | 8 (57%) | 3 (21%) |
| Sometimes | 11 (39%) | 4 (29%) | 7 (50%) |
| Always | 6 (21%) | 2 (14%) | 4 (29%) |
| **Reading is easy for me** |  |  |  |
| Never | 11 (38%) | 3 (20%) | 8 (57%) |
| Sometimes | 11 (38%) | 9 (60%) | 2 (14%) |
| Always | 7 (24%) | 3 (20%) | 4 (29%) |
| **Reading is important to me** |  |  |  |
| Never | 4 (14%) | 2 (13%) | 2 (14%) |
| Sometimes | 7 (24%) | 2 (13%) | 5 (36%) |
| Always | 18 (62%) | 11 (73%) | 7 (50%) |
| **I get words wrong when I read** |  |  |  |
| Never | 2 (7%) | 1 (7%) | 1 (7%) |
| Sometimes | 19 (66%) | 11 (73%) | 8 (57%) |
| Always | 8 (28%) | 3 (20%) | 5 (36%) |
| **Reading helps me keep in touch with people** |  |  |  |
| Never | 3 (10%) | 1 (7%) | 2 (14%) |
| Sometimes | 10 (34%) | 4 (27%) | 6 (43%) |
| Always | 16 (55%) | 10 (67%) | 6 (43%) |

Table S17: Follow-up SRS

|  | **Total (n = 31)** | **Intervention (n = 17)** | **Control (n = 14)** |
| --- | --- | --- | --- |
| **How confident are you in helping the person you support to read?** |  |  |  |
| 1 - Not at all confident | 0 (0%) | 0 (0%) | 0 (0%) |
| 2 | 0 (0%) | 0 (0%) | 0 (0%) |
| 3 | 2 (7%) | 0 (0%) | 2 (14%) |
| 4 | 4 (14%) | 4 (27%) | 0 (0%) |
| 5 | 5 (17%) | 0 (0%) | 5 (36%) |
| 6 | 10 (34%) | 6 (40%) | 4 (29%) |
| 7 - Very confident | 8 (28%) | 5 (33%) | 3 (21%) |
| **How difficult do you find it to help the person you support to understand the things they need to read?** |  |  |  |
| 1 - Very difficult | 2 (7%) | 2 (13%) | 0 (0%) |
| 2 | 2 (7%) | 2 (13%) | 0 (0%) |
| 3 | 3 (10%) | 1 (7%) | 2 (14%) |
| 4 | 8 (28%) | 4 (27%) | 4 (29%) |
| 5 | 7 (24%) | 2 (13%) | 5 (36%) |
| 6 | 6 (21%) | 3 (20%) | 3 (21%) |
| 7 - Not at all difficult | 1 (3%) | 1 (7%) | 0 (0%) |
| **To what extent do you feel that the things that you do to help the person you support to read have a positive effect?** |  |  |  |
| 1 – Have a very negative effect | 4 (14%) | 2 (13%) | 2 (14%) |
| 2 | 0 (0%) | 0 (0%) | 0 (0%) |
| 3 | 0 (0%) | 0 (0%) | 0 (0%) |
| 4 | 2 (7%) | 0 (0%) | 2 (14%) |
| 5 | 3 (10%) | 2 (13%) | 1 (7%) |
| 6 | 11 (38%) | 4 (27%) | 7 (50%) |
| 7 - Have a very positive effect | 9 (31%) | 7 (47%) | 2 (14%) |
| **How satisfied are you with the way in which you help the person you support to understand the things they need to read?** |  |  |  |
| 1 - Not satisfied at all | 1 (3%) | 1 (7%) | 0 (0%) |
| 2 | 2 (7%) | 1 (7%) | 1 (7%) |
| 3 | 1 (3%) | 0 (0%) | 1 (7%) |
| 4 | 6 (21%) | 2 (13%) | 4 (29%) |
| 5 | 5 (17%) | 2 (13%) | 3 (21%) |
| 6 | 8 (28%) | 4 (27%) | 4 (29%) |
| 7 - Very satisfied | 6 (21%) | 5 (33%) | 1 (7%) |

Table S18: ANCOVA Analysis

| **Outcome** | **Coefficient** | **95% CI** |
| --- | --- | --- |
| **DIBELS** |  |  |
| Letter naming fluency (LNF) | -1.28 | -6.79, 4.24 |
| Phonemic segmentation fluency (PSF) | -2.09 | -7.76, 3.58 |
| Correct letter sounds (CLS) | 1.64 | -4.49, 7.78 |
| Words read correctly (WRC) | -1.61 | -3.81, 0.59 |
| Word reading fluency (WRF) | -0.45 | -3.63, 2.73 |
| Words Correct | -8.50 | -16.46, -0.54 |
| Errors | -0.39 | -2.03, 1.25 |
| Accuracy | -0.10 | -0.28, 0.09 |
| Composite score | -3.55 | -8.96, 1.86 |
| **EQ5D** |  |  |
| Your health today | -1.75 | -20.62, 17.11 |
| Final EQ5D Index | 0.00 | -0.13, 0.12 |
| **PWI** |  |  |
| Raw score | 1.62 | -0.63, 3.87 |
| Final score | 6.73 | -2.64, 16.11 |

All models contain corresponding baseline score, setting and trial arm
